# New Insights into Diversity of Myanmarinidae (Hymenoptera: Apocrita), with Description of Two New Species from Mid-Cretaceous Myanmar Amber

**DOI:** 10.3390/insects17020147

**Published:** 2026-01-27

**Authors:** Zixiaocheng Wang, Yan Zheng, Alexandr P. Rasnitsyn, Ning Jia, Wenqian Wang, Liran Wang, Yaning Zhang, Feilong Zhao

**Affiliations:** 1 Institute of Geology and Paleontology, Linyi University, Shuangling Rd., Linyi 276000, China; 19853933818@163.com (Z.W.); wangwenqian2@lyu.edu.cn (W.W.); zhangyaning@163.com (Y.Z.); 2 Palaeontological Institute, Russian Academy of Sciences, Moscow 117647, Russia; alex.rasnitsyn@gmail.com; 3 Natural History Museum, London SW7 5BD, UK; 4 State Key Laboratory of Palaeobiology and Stratigraphy, Nanjing Institute of Geology and Palaeontology, East Beijing Road, Nanjing 210008, China; jianing@nigpas.ac.cn; 5 Lunan Technician College, Gaodu Rd., Linyi 276000, China; wangliran@163.com

**Keywords:** insect fossil, Myanmarinidae, taxonomy, mid-Cretaceous, burmese amber

## Abstract

Two new species assigned to the genus *Myanmarina* of the family Myanmarinidae are described and figured from male specimens preserved in Upper Cretaceous ambers from the Hukawng Valley of northern Myanmar. *Myanmarina simplex* sp. nov. can be distinguished by a small head, 13-segmented antennae, the first flagellomere equal to the second one in length and protibiae with a bifurcated spur. *Myanmarina grandis* sp. nov. is established based on a large head, 11-segmented antennae, the first flagellomere shorter than the second one and all tibiae with a spur and dorso-apical tooth. In addition, the key to known species of *Myanmarina* was updated.

## Simple Summary

Hymenoptera are well represented in the fossil record, particularly in the mid-Cretaceous amber from northern Myanmar, representing a geographic isolation and endemism. Two new fossil taxa of the extinct family Myanmarinidae, *Myanmarina simplex* sp. nov. and *M. grandis* sp. nov., are described and illustrated from mid-Cretaceous Kachin amber. The notable endemic characters revealed are summarized for comparison; they also suggest the high species-level diversity of Myanmarinidae.

## 1. Introduction

Cretaceous amber from Myanmar has preserved the amazing paleodiversity of insects and particularly that of Hymenoptera, with over 382 species in 216 genera across 71 families [1,2]. Interestingly, 36 hymenopteran families have only recently been discovered from the Burmese (Myanmar) amber, providing unique insights into the Cretaceous entomofauna [2]. The family Myanmarinidae, an extinct lineage of parasitoid wasps, was famous as endemic to Myanmar terrain in the mid-Cretaceous [3,4,5,6] and is placed with one relict family, Stephanidae, and four extinct families, Aptenoperissidae, Ephialtitidae, Ohlhoffiidae and Myanmarinidae, in the superfamily Stephanoidea [3,4,7,8,9,10,11,12]. It is morphologically characterized by a straight propodeum in the side view (lacking a posterior slope), so a real constriction is absent between the propodeum and the metasoma [4]. Furthermore, myanmarinid wasps are easily distinguishable from all other Hymenoptera in having a small body (below 5 mm in length), a narrow, cylindrical head that is smooth without a crown, oligomereous antenna (up to 14-segmented), extremely reduced venation and tibiae with a single apical spur and dorso-apical tooth [4,6].

Myanmarinidae were first established in 2018 by Zhang and Rasnitsyn on the basis of five male specimens of three species within the single genus *Myanmarina*, *M. lisu* Zhang and Rasnitsyn, 2018, *M. kachin* Zhang and Rasnitsyn, 2018 and *M. lahu* Zhang and Rasnitsyn, 2018 [4]. Subsequently, *M*, *jeannineae* Li et al., 2018, was reported based on one female and three male specimens, with integrally compared morphological features from the male and female specimens, showing sexual dimorphism such as the size of the body and the number of antennomeres [3,13]. Additionally, *M. sidorchukae* Jouault et al., 2020, was recorded based on an exquisitely preserved specimen, and the key to known species of *Myanmarina* males was provided [5]. Recently, *M. diversa* Zheng, Zhang & Rasnitsyn, 2022, was confirmed as the sixth myanmarinid wasp, and the emended diagnosis of Myanmarinidae was proposed by re-examination and revision [6]. Although this family is extraordinary and only discovered from the Cretaceous in Burmese amber of insular origin, hitherto six species within just a single genus have been discovered, exhibiting high morphological and taxonomic diversity [4,6].

Herein we collected two well-preserved fossil specimens of Myanmarinidae from the mid-Cretaceous Burmese amber in Kachin, northern Myanmar. Furthermore, we propose two new species, *Myanmarina simplex* sp. nov. and *M. grandis* sp. nov. described and illustrated in this paper. Our new findings of these new species provide more comprehensive evidence for the basal morphological variation and detailed analysis of the taxonomy of myanmarinid wasps and enhance our understanding of endemic hymenopteran fauna in Myanmar amber.

## 2. Materials and Methods

The type specimens described in this study were collected form the Hukawng Valley of Kachin State (26°20′ N, 96°36′ E), northern Myanmar (detailed map in Grimaldi and Ross, 2017). Radiometric analyses established the earliest Cenomanian 98.79 ± 0.62 Ma for the Kachin amber according to U-Pb zircon data of the volcanoclastic matrix found within the amber enclosing sediments [14]. Additionally, an earlier dating based on ammonites supports a late Albian–early Cenomanian age from the amber-bearing deposits [15]. However, the true age of the amber deposit by palaeontological and zircon means remains to be resolved [9,16].

All type specimens reported herein were collected legally prior to 2015 without any armed conflict and ethnic strife in Myanmar and were permanently housed in the Institute of Geology and Paleontology, Linyi University. The two amber specimens were re-cut and polished to as flat as was possible with surfaces parallel to the inclusion by the precision diamond-wire saw (Chassieu, France) and rotary polisher (Paris, France). The samples were examined and photographed with a Nikon SMZ-10R stereoscopic microscope (Shanghai, China) and VHX 5000 digital microscope platform (Lakewood, CO, USA). The line drawings were composed with CorelDraw 2020 (https://www.coreldraw.com/en/, accessed on 1 January 2020) and Adobe Photoshop CS6 (https://www.adobe.com/, accessed on 24 April 2012). Measurements were established with Image J software v.1.8.0 (National Institutes of Health, Bethesda, MD, USA). The morphological terminology of body and wing venation used herein follows [4,6].

## 3. Results

Systematic palaeontology

Order Hymenoptera Linnaeus, 1758

Suborder Apocrita Gerstaecker, 1867

Superfamily Stephanoidea Leach, 1815

Family Myanmarinidae Zhang and Rasnitsyn, 2018

Genus *Myanmarina* Zhang and Rasnitsyn, 2018

Type species: *M. lisu* Zhang and Rasnitsyn, 2018;

Included species: *M. kachin* Zhang and Rasnitsyn, 2018; *M. lahu* Zhang and Rasnitsyn, 2018; *M. jeannneae* Li, Shih, Rasnitsyn and Ren, 2018; *M. sidorchukae* Jouault, Rasnitsyn and Perrichot, 2020; *M. diversa* Zheng and Rasnitsyn, 2022; *M. simplex* Zheng, sp. nov., and *M. grandis* Zheng, sp. nov.

**Figure 1:**
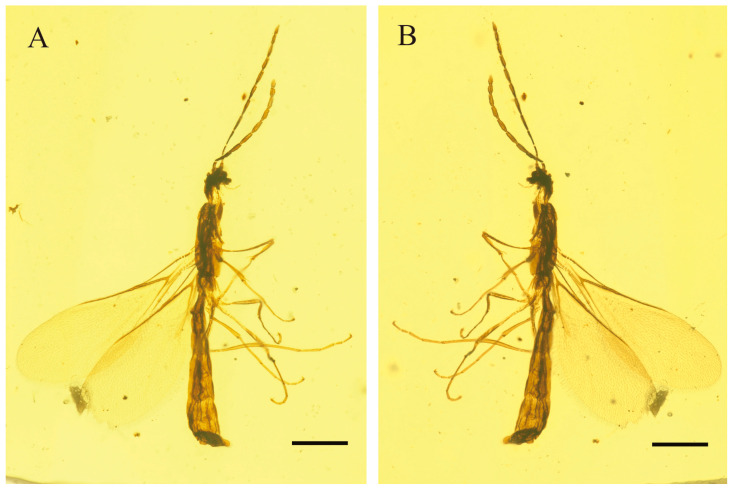
Photographs of *Myanmarina simplex* sp. nov., holotype, LYU-HY-2032. (**A**), habitus in left lateral view; (**B**), habitus in right lateral view. Scale bars = 0.5 mm.

**Figure 2:**
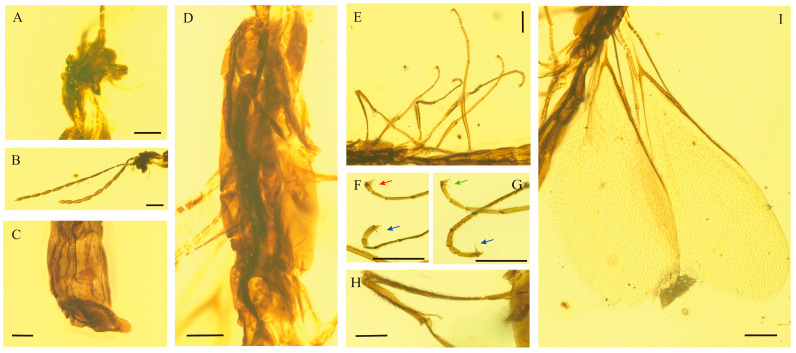
Photograph of *Myanmarina simplex* sp. nov., holotype, LYU-HY-2032. (**A**), head; (**B**), antenna; (**C**), male genitalia; (**D**), mesosoma; (**E**), legs; (**F**), claws of protarsi and mesotarsi (red arrow shows protarsi and blue arrow shows mesotarsi); (**G**), claws of mesotarsi and metatarsi (green arrow shows metatarsi and blue arrow shows mesotarsi); (**H**), spur of protibia; (**I**), forewings. Scale bars: (**A**,**C**,**I**) = 0.1 mm, (**B**,**D**–**H**) = 0.2 mm.

**Figure 3:**
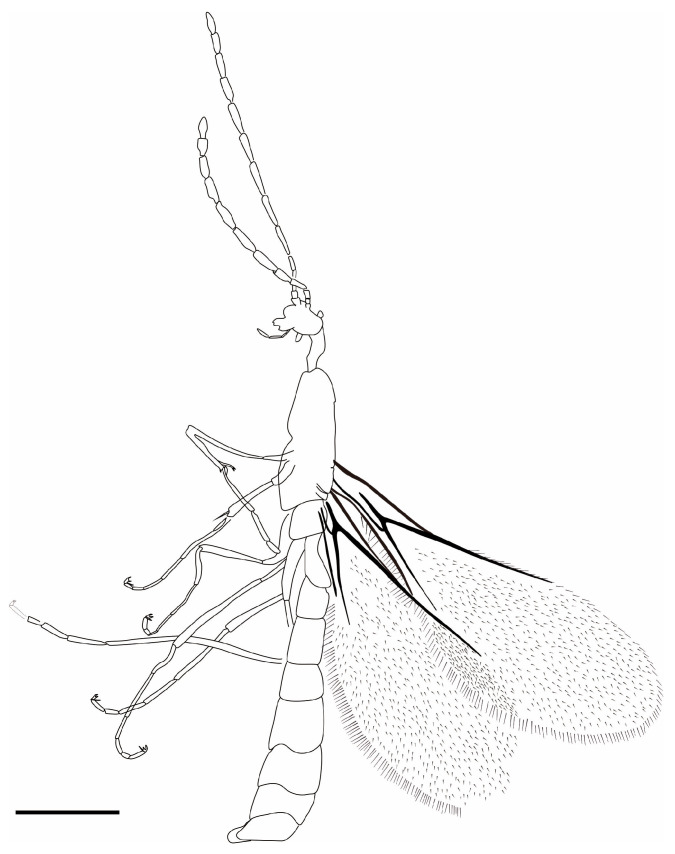
Line drawing *Myanmarina simplex* sp. nov. Scale bar = 0.5 mm.

*Myanmarina simplex* Zheng, sp. nov. (Figure 1, Figure 2 and Figure 3)

urn:lsid:zoobank.org:pub:3FF4BE73-17EB-4944-A317-EE544B3785E1

Etymology. The specific name is derived from the Latin word “simplex”, meaning simple, referring to the dramatically reduced forewing venation, with only a few veins present in the specimen.

Type material. Holotype, male, LYU-HY-2032, mid-Cretaceous Burmese amber, housed in the Institute of Geology and Paleontology, Linyi University; well-preserved.

Locality and horizon. Hukawng Village, Kachin State, northern Myanmar; mid-Cretaceous (upper Albian to lower Cenomanian).

Diagnosis. Small head, long and round. Antenna with 13 antennomeres, with the first and second flagellomeres subequal in length, apical segments gradually shortening. Long maxillary palp. Protibia with bifurcated spur. Hind wing with no basal membrane of hamuli.

Description. Dark reddish brown integument as preserved, with paler flagellomeres, wings and legs. Body length 2.76 mm, from top of head to end of metasoma.

Head, long elliptic, 0.17 mm long and 0.12 mm high. Compound eye, not easily recognizable. Ocelli difficult to discern. Clypeus transverse, with semilunar anterior margin. Mandible, short. Maxillary palps, slender and long, with four segments visible, basal palpomeres, short, apical three, narrow and elongate, 0.02 mm, 0.04 mm, 0.4 mm and 0.07 mm in length, respectively. Antennae, long and filiform, 13-segmented, about 1.54 mm in length and 0.04 mm in maximum width, inserted moderately low on the head in lateral view, densely covered with setae and sensilla. Scape, cylindrical and remarkably swollen, more robust than remaining antennomeres, about 1.50 times the length and 1.25 times the width of the pedicel; pedicel, short and broad, 0.04 mm in length and 0.03 mm in width; flagellomeres, slender, about 1.60 times to 5.50 times longer than wide. First flagellomere distinctly narrower than pedicel, with length 0.15 mm and width 0.02 mm and nearly as long as the second one; third to seventh flagellomeres conspicuously longer than other flagellomeres in length; remaining flagellomeres gradually shorter toward apex. Apicalmost flagellomere oval. Lengths of flagellomeres (in mm): 0.07, 0.07, 0.14, 0.15, 0.17, 0.13, 0.14, 0.11, 0.12, 0.08 and 0.11.

Mesosoma subcylindrical, length 0.78 mm and height 0.18 mm, much wider than head in lateral view. Pronotum, short and prominent. Propleura, long, forming distinct neck. Mesonotum, large, with details obscure. Mesoscutellum, convex in profile, and 0.13 mm long. Metanotum, short, 0.03 mm long; metapostnotum, open and long. Propodeum, convex, with anterior face almost as high as metanotum and posterior face conspicuously higher than metanotum.

Forewing, long and narrow, about 0.76 mm in length and 0.17 mm in width, densely pubescent and with extraordinary long marginal setae along posterior margin. Wing venation dramatically reduced, only a few veins present, mainly in basal area. Pterostigma undeveloped. Vein C and R fused, C+R tubular and thickened, more than half of forewing in length. 1-Rs and 1-M fused and aligned as very thick Rs & M, longer than M+Cu. M+Cu formed an obtuse angle with Rs & M. 1-Cu short, nearly one-sixth as long as Rs & M. Crossvein cu-a aligned with 1A, meeting Cu distinctly distal to M+Cu apex. Free Cu long and nebulous, subparallel to posterior margin and practically fading. All distal wing surface free of veins. Hind wings with only R and long posterior setae present, with membrane rudiment present only distal to hamuli (apparently two present).

Legs, elongate, slender and covered with short appressed setae. Forelegs with femur distinctly longer than tibia; tibia with bifurcated spur and a dorso-apical tooth; tarsus with five tarsomeres, basitarsus shorter than remaining tarsomeres combined; measurements (mm) of femur, tibia and five tarsomeres as follows: 0.44, 0.29, 0.30, 0.14, 0.11, 0.06 and 0.05, respectively. Mid legs relatively short; coxa large and elongate, 0.29 mm in length and 0.07 mm in width as preserved; trochanter 0.07 mm in length; femur 0.27 mm long and 0.04 mm wide; tibia shorter than fore- and metatibia, 0.19 mm long and 0.03 mm wide; tarsus with five tarsomeres, first tarsomere distinctly longer than others, and fourth tarsomere shortest; tarsomere length 0.24 mm, 0.11 mm. 0.07 mm, 0.04 mm and 0.07 mm. Hind legs, long; coxa and trochanter poorly preserved; femur, slender (ca. 0.36 mm long and 0.04 mm wide), with long, straight apical spur; tibia longer than fore- and mesotibia; tarsal segment ratio I: II: III: IV: V = 0.38: 0.19: 0.15: 0.06: 0.09. All pretarsal claws simple and acute, with straight preapical tooth.

Metasoma, oblong with eight segments, 1.63 mm in length, 0.27 mm in maximum width. First metasomal segment, trapezoid, with dorsal surface concave, much narrower at base in dorsal view, 0.26 mm long, 0.13 mm maximum width and 0.06 mm minimum width; second to seventh metasomal segments almost equal in length; third to fifth metasomal segments gradually widened from base to apical apex; sixth metasomal segment widest, 0.23 mm long and 0.27 mm wide; the following seventh and eighth segments apparently narrower than previous ones. Male genitalia well preserved but overlapped, claspers wide and long, covered with dense setae triangular in side view; gonostylus broadens apically; penis valve with apex proportionally flat and covered with long setae; otherwise, genitalia not visible.

**Figure 4:**
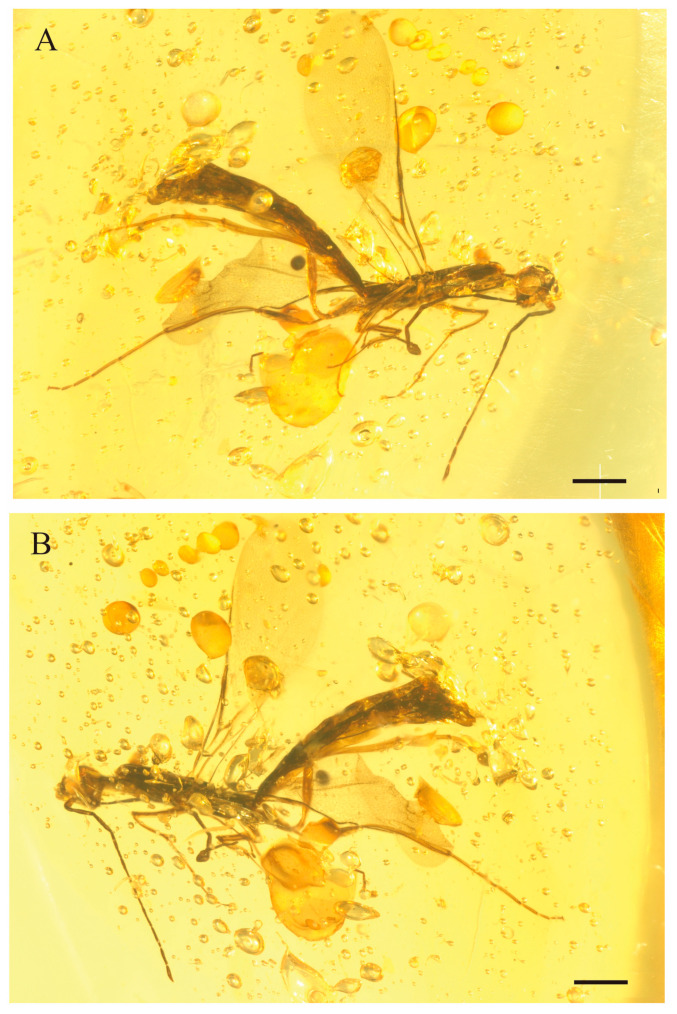
Photographs of *M. grandis* sp. nov., holotype, LYU-HY-2035. (**A**), habitus in left view; (**B**), habitus in right view. Scale bars = 0.5 mm.

**Figure 5:**
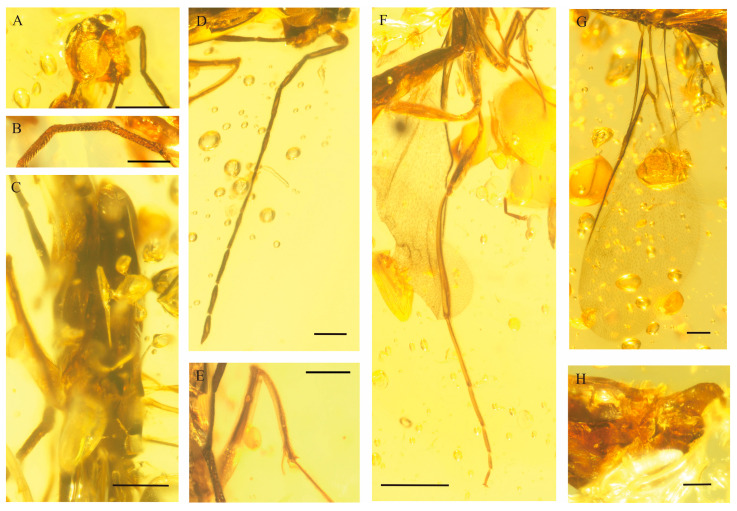
Photographs of *Myanmarina simplex* sp. nov., holotype, LYU-HY-2035. (**A**), head; (**B**), multiparous plate sensilla of flagellar segments; (**C**), mesosoma; (**D**), antenna; (**E**), spure of protibia; (**F**), hind leg; (**G**), forewing and hindwing; (**H**), male genitalia. Scale bars: (**A**–**C**), (**E**–**H**)= 0.2 mm, (**D**) = 0.1 mm.

**Figure 6:**
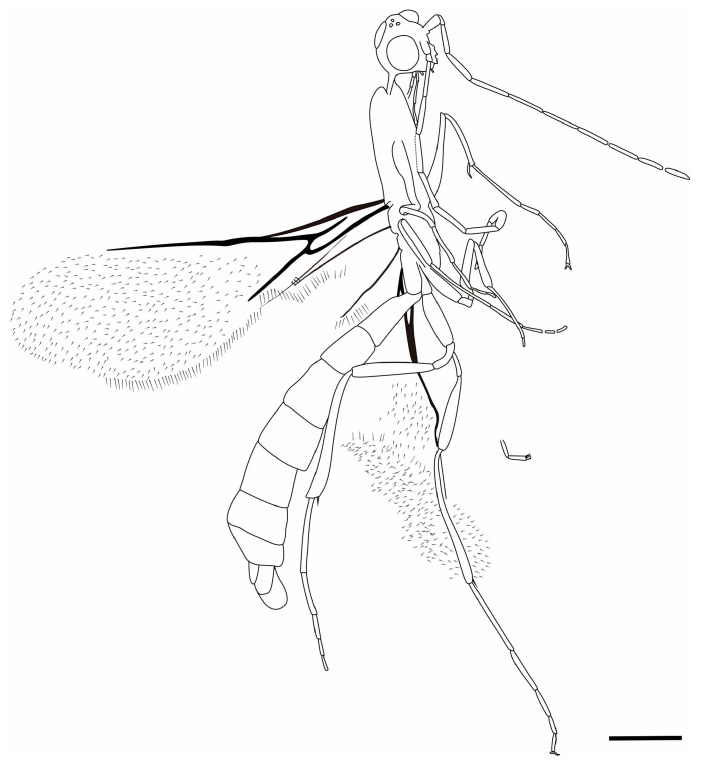
Line drawing *M. grandis* sp. nov. Scale bar = 0.5 mm.

*Myanmarina grandis* Zheng, sp. nov. (Figure 4, Figure 5 and Figure 6)

urn:lsid:zoobank.org:pub:F419FEDA-BDB6-4D13-9EF2-72320877C8E8

Etymology. The specific name “grandis”(Latin), means large, referring to the body size, compared with other species of the *Myanmarina*.

Type material. Holotype, male, LYU-HY-2035; mid-Cretaceous Burmese amber, housed in Institute of Geology and Paleontology, Linyi University; well-preserved.

Locality and horizon. Hukawng Village, Kachin State, northern Myanmar; mid-Cretaceous (upper Albian to lower Cenomanian).

Diagnosis. Head large and oval, with vertex flat to prominent convex. Antenna with 11 antennomeres, scape about 1.5 times as long as pedicel, first flagellomere shorter than second; maxillary palps long; tibiae with spur and not distinctly bifid.

Description. Integument, dark reddish brown as preserved, with scape, pedicel, flagellomeres, tibiae and tarsi less dark. Male, body length 2.65 mm, excluding antennae.

Head, subglobose, slightly shorter than wide in lateral view, with length 0.45 mm and height 0.34 mm. Compound eye, large and ovoid, with height 0.25 mm and width 0.22 mm. Ocelli, present and small, situated in a triangle on top of vertex above compound eyes. Prominent facial convexity projecting length 0.10 mm and width 0.07 mm in profile. Maxillary palps slightly longer than height of head, with four segments visible, basal palpomeres short and broad, two apical ones clavate and elongate visible, 0.06 mm, 0.10 mm, 0.16 mm and 0.18 mm long, respectively. Antenna with 11 antennomeres, 2.33 mm long and longer than head and mesosoma combined, inserted moderately low on head in lateral view and covered with setae and sensilla. Scape distinctly robust and broad, almost 1.50 times the length and width of pedicel; pedicel short and narrow, 0.06 mm long and 0.04 mm wide; flagellomeres much longer than wide; first flagellomere slightly shorter than the second one; flagellomere II to V equal in length, remaining flagellomeres gradually shortening toward apex. Apicalmost flagellomere, pointed oval. Lengths of flagellomeres (in mm): 0.19, 0.24, 0.28, 0.26, 0.26, 0.22, 0.23, 0.18 and 0.17.

Mesosoma, elongate and column-shaped, more slender than head in lateral view, 1.13 mm long and 0.28 mm high. Pronotum, smooth and short, with no details visible. Propleura, large, almost abut in middle third; mesonotum difficult to observe laterally. Mesoscutellum 0.18 mm long. Metanotum short, 0.10 mm long. Propodeum concave in posterior margin and nearly 1.6 times as long as mesonotum.

Forewing, clear and hyaline, ca. 2.76 mm long and 0.90 mm wide, densely pubescent and with particularly long marginal setae inserted in distal half of posterior margin. Venation, brown to light brown as preserved, highly reduced, mainly most basal veins presented. Pterostigma absent. Vein, C+R thick, about half length of wing; Rs & M strongly oblique and widening toward apical wing margin, almost equal to M+Cu in length. M+Cu absent basally but form an obtuse angle with Rs & M. 1-Cu short, nearly one-third length of Rs & M. cu-a present but very short, aligned with 1A. Free Cu long, nebulous, subparallel to posterior wing margin and fading distally. Hind wing short and narrow, without wing venation, but R, three hamuli and narrow membrane distal to hamuli, with long posterior setae present.

Legs, slender, bearing short and thick setae. Fore femur (ca. 0.61 mm long and 0.09 mm wide) fusiform and pronouncedly longer and basally wider than tibia; foretibia 0.45 mm long and 0.04 mm wide, with elongated and curved spur not distinctly split apically and with velum reaching its apex and with dorso-apical tooth; tarsus with five tarsomeres, basitarsus longer than tibia and shorter than remaining tarsomeres combined; measurements (mm) of tarsomeres as follow: 0.42 mm, 0.12 mm, 0.15 mm, 0.12 mm and 0.10 mm, respectively. Mesocoxa, 0.27 mm in length and 0.10 mm in width; mesotrochanter, 0.09 mm long and 0.06 mm wide; mesofemur, (ca. 0.43 mm long) columniform, as wide as mesotibia; mesotibia, wider and shorter than protibia, ca. 0.37 mm long and 0.06 mm wide; mesotarsus with five tarsomeres, measurements as follow: 0.47 mm, 0.26 mm, 0.15 mm, 0.10 mm and 0.05 mm. Metacoxa, elongate and swollen, with 0.45 mm long and 0.09 mm wide; metatrochanter, longer, 0.22 mm long and 0.05 mm wide; metafemur, nearly twice as long as profemur, 0.52 mm long and 0.11 mm wide; metatarsomeres, length 0.53 mm, 0.33 mm, 0.199 mm, 0.12 mm and 0.12 mm. Apex of all tibiae with long spur and short dorsoapical tooth directed posteriorly; tarsal claws simple and bent, arolium and preapical tooth visible in meso- and metapretarsi.

Metasoma elongated, with eight external tergites, 2.69 mm in length and 0.36 mm in maximum width. First metasomal segment, slender and subcylindrical, 0.37 mm long and 0.12 mm wide; second metasomal segment, longest, third to sixth metasomal segments, almost equal in length, seventh to eighth metasomal segments, shorter and wider than former segments. Claspers of male genitalia, long and wide; gonostylus broadens apically; penis valve short and visible.

Revised key to males of the known species of *Myanmarina*.

1. Antenna 11-segmented……………..…..………………………………………..………2

– Antenna 12- or 13-segmented……..…..…………………..…..………...……………….4

2. First flagellomere short, obviously shorter than second. ……………………………………………………………………*M. grandis* Zheng sp. nov.

– First flagellomere twice as long as second.……………………………………………3

3. Metafemur long and slender (more than 4× as long as wide), male claspers very long (as long as or longer than preceding metasomal segment) ……………………………………………………….*M. lisu* Zhang and Rasnitsyn, 2018

– Metafemur short and thick (3× as long as wide), metacoxa and male claspers short (shorter than preceding metasomal segment) ………*M. kachin* Zhang and Rasnitsyn, 2018

4. Antenna 12-segmented………………………….……………………………………….5

– Antenna 13-segmented………………………….………………………………………..6

5. Metafemur thin, only slightly wider medially, about as long as metatibia……………………………………………………….*M. lahu* Zhang and Rasnitsyn, 2018

– Metafemur thick, conspicuously wider medially, much shorter than metatibia… 7

6. Head elongated, much longer than wide in dorsal view …………………………………………………………….…….*M. jeannineae* Li et al., 2018

– Head short, rounded, slightly longer than wide in dorsal view……...................................................................................*M. sidorchukae* Jouault et al., 2020

7. Metafemur conspicuously wider medially, three apical antennomeres of subequal length, protibial spur bifid…………………….…*M. diversa* Zheng, Zhang & Rasnitsyn 2022

– Metafemur thin, apical antennomeres distinctly gradually shortening, protibial spur acute………………………………………………………………………*M. simplex* Zheng sp. nov.

## 4. Discussion

The mid-Cretaceous Burmese amber is one of the largest and richest fossil insect Lagerstätten known [1,17,18,19]. Hitherto, 384 hymenopteran species out of 2233 described insect species reveal a dramatic and amazing diversity of fossil insects preserved in mid-Cretaceous amber from Myanmar [2,4,6,9]. Noticeably, Myanmarinidae were described by Zhang et al., 2018 and amended by Zheng et al., 2022 showing highly specialized morphological characters [3,4,5,6,13].

Our fossils possess particular characters easily recognized as Myanmarinidae among the Stephanoidea as evidenced by a small and slender body size, oligomerous antenna, a straight propodeum, without constriction between propodeum and metasoma, deeply reduced wing venation and a single tibial spur [3,4]. Until now, the fossil record of this family has only one genus, *Myanmarina,* with six species [3,4,5,6], and the diversity and character distribution among the identified fossil species of Myanmarinidae are shown in Table 1. We herein report the discovery of two interesting species, *Myanmarina simplex* sp. nov. and *M. grandis* sp. nov., preserved in mid-Cretaceous amber from Myanmar. *Myanmarina simplex* sp. nov. can be distinguished from other species of the genus as follows: it differs from *M. kachin*, *M. lisu*, *M. jeannineae*, *M. sidorchukae* and *M. lahu* by 13-segmented antenna; differs from *M. diversa* by the first flagellomere being nearly equal to the second one and having a slender metafemur. *Myanmarina grandis* sp. nov. differs from other species primarily by its 11-segmented antenna (vs. 12-segmented in *M. lahu*, *M. jeannineae* and *M. sidorchukae*; 13-segmented in *M. simplex* sp. nov. and *M. diversa*). Despite the considerable similarity between *M. grandis* sp. nov. *M. lisu* and *M. kachin*, it is not difficult to distinguish this new species from the latter two by a number of morphological details of the antenna, such as the first flagellomere being as long as the second one (vs. the first flagellomere being extremely long, almost twice length of the second one in *M. lisu* and *M. kachin*).

It is interesting that the two newly described *Myanmarina* fossils significantly extend the known record of myanmarinid wasps from the Cretaceous, as well as considerably increase our knowledge concerning the diversity of these unusual wasps. Noteworthily, *Myanmarina simplex* sp. nov. and *M. grandis sp*. nov. are endemic characters of the Burmese amber biota emphasizing high species-level diversity and bizarre morphology, with an astonishing diversity of hymenopteran taxa in the fossil record in the mid-Cretaceous.

## 5. Conclusions

*Myanmarina simplex* sp. nov. and *M. grandis* sp. nov. are assigned herein to the family Myanmarinidae, which is only known from mid-Cretaceous Kachin amber as the seventh and eighth species of a single genus *Myanmarina*, representing a distinctive body of morphological features, particularly the extremely reduced forewing venation pattern and highlighting great diversity of the parasitoid hymenopterans in the Cretaceous insect faunas. Additionally, these new and important species increase the number of endemic taxa resulting from isolation of the West Burma Plate.

## Figures and Tables

**Table 1:** Diversity and character distribution among the identified fossil species of Myanmarinidae.

	Characters										Reference
	MNF	SP	F1	F1–F2	MP	PFT	MOFT	MEFT	MF	1-Cu	
*Myanmarina lisu* Zhang and Rasnitsyn, 2018	0	0	1	2	1	0	0	0	0	-	[4]
*Myanmarina kachin* Zhang and Rasnitsyn, 2018	0	1	0	2	1	0	0	1	1	0	[6]
*Myanmarina lahu* Zhang and Rasnitsyn, 2018	1	1	1	1	0	1	1	0	0	1	[4]
*Myanmarina jeannineae* Li et al., 2018	1	1	1	1	2	1	1	0	0	2	[3]
*Myanmarina sidorchukae* Jouault, Rasnitsyn & Perrichot, 2020	1	1	0	1	2	1	0	0	0	1	[5]
*Myanmarina diversa* Zheng, Zhang & Rasnitsyn, 2022	2	1	1	2	0	1	1	0	0	0	[6]
*Myanmarina simplex* sp. nov.	2	1	1	1	2	1	1	0	1	0	This study
*Myanmarina grandis* sp. nov.	0	1	1	0	2	1	1	0	1	2	This study

MNF = antennal thread of male, 11-segmented (0) or 12-segmented (1) or 13-segmented (2); SP = scape as long as pedicel (0) or scape longer than pedicel (1); F1 = first flagellomere, curved (0) or straight (1); F1–F2 = first flagellomere shorter than second (0) or as long as (1) or longer (2); MP = maxillary palps shorter than head height (0) or as long as (1) or longer (2); PFT = profemur shorter than protibial in length (0) or loner (1); MOFT = mesofemur shorter than mesotibia in length (0) or longer (1); MEFT = metafemur shorter than metatibia in length (0) or longer (1); MF = metafemur swollen (0) or slender (1); 1-Cu = 1-cu shorter than one-fourth length of Rs & M (0) or as long as (1) or longer (2).

## Data Availability

All data from this study are available in this paper and the associated papers. All the specimens are housed in the Institute of Geology and Paleontology, Linyi University, Linyi, China.

## References

[1] Ross A.J. (2024). Complete checklist of Burmese (Myanmar) amber taxa 2023. Mesozoic.

[2] Ross A.J. (2025). Supplement to the Burmese (Myanmar) amber checklist and bibliography, 2024. Palaeoentomology.

[3] Li L.F., Shih C., Rasnitsyn A.P., Li D., Ren D. (2018). A new wasp of Myanmarinidae (Hymenoptera: Stephanoidea) from the mid-Cretaceous Myanmar amber. Cretac. Res..

[4] Zhang Q., Rasnitsyn A.P., Wang B., Zhang H.C. (2018). Myanmarinidae, a new family of basal Apocrita (Hymenoptera: Stephanoidea) from mid-Cretaceous Burmese amber. Cretac. Res..

[5] Jouault C., Rasnitsyn A.P., Perrichot V. (2020). A new myanmarinid wasp (Hymenoptera: Stephanoidea) from mid-Cretaceous Burmese amber. Cretac. Res..

[6] Zheng Y., Hu H.Y., Wang H., Chen J., Zhang Q., Zhang H.C., Rasnitsyn A.P. (2022). *Myanmarina diversa* sp. nov (Hymenoptera, Myanmarinidae) from mid-Cretaceous Kachi amber, northern Myanmar. Cretac. Res..

[7] Engel M.S., Grimaldi D.A., Ortega-Blanco J. (2013). A stephanid wasp in midcretaceous Burmese amber (Hymenoptera: Stephanidae), with comments on the antiquity of the hymenopteran radiation. J. Kans. Entomol. Soc..

[8] Rasnitsyn A.P., Poinar G., Brown A.E. (2017). Bizzare wingless parasitic wasp from mid-Cretaceous Burmese amber (Hymenoptera, Ceraphronoidea, Aptenoperissidae fam. nov.). Cretac. Res..

[9] Rasnitsyn A.P., Öhm-Kühnle C. (2018). Three new female *Aptenoperissus* from mid-Cretaceous Burmese amber (Hymenoptera, Stephanoidea, Aptenoperissidae): Unexpected diversity of paradoxical wasps suggests insular features of source biome. Cretac. Res..

[10] Zhang Q., Rasnitsyn A.P., Wang B., Zhang H.C. (2018). New data about the enigmatic wasp from mid-cretaceous Burmese amber (Hymenoptera, Stephanoidea, Aptenoperissidae). Cretac. Res..

[11] Rasnitsyn A.P., Ansorge J. (2000). Two new Lower Cretaceous hymenopterous insects (Insecta: Hymenoptera) from Sierra del Montsec, Spain. Palaontol. Z..

[12] Jouault C., Rasnitsyn A.P., Perrichot V. (2021). Ohlhoffiidae, a new Cretaceous family of basal parasitic wasps (Hymenoptera: Stephanoidea). Cretac. Res..

[13] Li L.F., Shih C., Rasnitsyn A.P., Yang T., Gao Y., Ren D. (2022). Two new wasps from mid-Cretaceous Myanmar amber (Hymenoptera: Apocrita). Cretac. Res..

[14] Shi G.H., Grimaldi D.A., Harlow G.E., Jing W., Wang J., Yang M.C., Lei W.Y., Li Q., Li X.H. (2012). Age constraint on the Burmese amber based on UePb dating of Zircons. Cretac. Res..

[15] Yu T.T., Kelly R., Mu L., Ross A.J., Kennedy J., Broly P., Xia F.Y., Zhang H.C., Wang B., Dilcher D. (2019). An ammonite trapped in Burmese amber. Proc. Natl. Acad. Sci. USA.

[16] Smith R.D.A., Ross A.J. (2018). Amberground pholadid bivalve borings and inclusions in Burmese amber: Implications for proximity of resin-producing forests to brackish waters, and the age of the amber. Earth Environ. Sci. Trans. R. Soc. Edinb..

[17] Zheng Y., Hu H.Y., Zhang H.C., Chen J., Rasnitsyn A.P., Zhuo D. (2021). New genus and species of syspastoxyelid sawflies (Insecta, Hymenoptera) from the mid-Cretaceous Kachin amber with a review of the family Syspastoxyelidae. Cretac. Res..

[18] Zheng Y., Chen J., Zhang H.C., Rasnitsyn A.P. (2021). New angarosphecid wasp (Hymenoptera: Apoidea, Angarosphecidae) from the mid-Cretaceous Burmese amber. Cretac. Res..

[19] Wang Z.Q., Zhuo D., Wang H., Chen J., Zhang H.C., Rasnitsyn A.P., Zheng Y. (2025). New anaxyelid woodwasps (Hymenoptera: ’Symphyta’: Anaxyelidae) from Mid-Cretaceous Kachin amber. Alcheringa.

